# Three-quarters attack rate of SARS-CoV-2 in the Brazilian Amazon during a largely unmitigated epidemic

**DOI:** 10.1126/science.abe9728

**Published:** 2020-12-08

**Authors:** Lewis F. Buss, Carlos A. Prete, Claudia M. M. Abrahim, Alfredo Mendrone, Tassila Salomon, Cesar de Almeida-Neto, Rafael F. O. França, Maria C. Belotti, Maria P. S. S. Carvalho, Allyson G. Costa, Myuki A. E. Crispim, Suzete C. Ferreira, Nelson A. Fraiji, Susie Gurzenda, Charles Whittaker, Leonardo T. Kamaura, Pedro L. Takecian, Pedro da Silva Peixoto, Marcio K. Oikawa, Anna S. Nishiya, Vanderson Rocha, Nanci A. Salles, Andreza Aruska de Souza Santos, Martirene A. da Silva, Brian Custer, Kris V. Parag, Manoel Barral-Netto, Moritz U. G. Kraemer, Rafael H. M. Pereira, Oliver G. Pybus, Michael P. Busch, Márcia C. Castro, Christopher Dye, Vítor H. Nascimento, Nuno R. Faria, Ester C. Sabino

**Affiliations:** 1 Departamento de Molestias Infecciosas e Parasitarias and Instituto de Medicina Tropical da Faculdade de Medicina da Universidade de São Paulo, São Paulo, Brazil.; 2 Departamento de Engenharia de Sistemas Eletrônicos, Escola Politécnica da Universidade de São Paulo, São Paulo, Brazil.; 3 Fundação Hospitalar de Hematologia e Hemoterapia do Amazonas, Manaus, Brazil.; 4 Fundação Pró-Sangue–Hemocentro de São Paulo, São Paulo, Brazil.; 5 Laboratório de Investigação Médica em Patogênese e Terapia dirigida em Onco-Imuno-Hematologia (LIM-31), Departamento de Hematologia, Hospital das Clínicas HCFMUSP, Faculdade de Medicina da Universidade de São Paulo, São Paulo, Brazil.; 6 Fundação Hemominas–Fundação Centro de Hematologia e Hemoterapia de Minas Gerais, Belo Horizonte, Brazil.; 7 Faculdade Ciências Médicas de Minas Gerais, Belo Horizonte, Brazil.; 8 Department of Virology and Experimental Therapy, Institute Aggeu Magalhaes, Oswaldo Cruz Foundation, Recife, Brazil.; 9 Department of Global Health and Population, Harvard T. H. Chan School of Public Health, Boston, MA, USA.; 10 Department of Infectious Disease Epidemiology, School of Public Health, Imperial College London, London, UK.; 11 Institute of Mathematics and Statistics, University of São Paulo, São Paulo, Brazil.; 12 Center of Mathematics, Computing and Cognition–Universidade Federal do ABC, São Paulo, Brazil.; 13 Oxford School of Global and Area Studies, Latin American Centre, University of Oxford, Oxford, UK.; 14 Vitalant Research Institute, San Francisco, CA, USA.; 15 University of California, San Francisco, CA, USA.; 16 MRC Centre for Global Infectious Disease Analysis, J-IDEA, Imperial College London, London, UK.; 17 Instituto Gonçalo Moniz–Fundação Oswaldo Cruz (Fiocruz), Salvador, Brazil.; 18 Department of Zoology, University of Oxford, Oxford, UK.; 19 Institute for Applied Economic Research–Ipea, Brasília, Brazil.

## Abstract

Severe acute respiratory syndrome coronavirus 2 (SARS-CoV-2) spread rapidly in Manaus, the capital of Amazonas state in northern Brazil. The attack rate there is an estimate of the final size of the largely unmitigated epidemic that occurred in Manaus. We use a convenience sample of blood donors to show that by June 2020, 1 month after the epidemic peak in Manaus, 44% of the population had detectable immunoglobulin G (IgG) antibodies. Correcting for cases without a detectable antibody response and for antibody waning, we estimate a 66% attack rate in June, rising to 76% in October. This is higher than in São Paulo, in southeastern Brazil, where the estimated attack rate in October was 29%. These results confirm that when poorly controlled, COVID-19 can infect a large proportion of the population, causing high mortality.

## Attack rate in Manaus

Severe acute respiratory syndrome coronavirus 2 (SARS-CoV-2) incidence peaked in Manaus, Brazil, in May 2020 with a devastating toll on the city's inhabitants, leaving its health services shattered and cemeteries overwhelmed. Buss *et al.* collected data from blood donors from Manaus and São Paulo, noted when transmission began to fall, and estimated the final attack rates in October 2020 (see the Perspective by Sridhar and Gurdasani). Heterogeneities in immune protection, population structure, poverty, modes of public transport, and uneven adoption of nonpharmaceutical interventions mean that despite a high attack rate, herd immunity may not have been achieved. This unfortunate city has become a sentinel for how natural population immunity could influence future transmission. Events in Manaus reveal what tragedy and harm to society can unfold if this virus is left to run its course.

*Science*, this issue p. 288; see also p. 230

## Teaser

The spread of COVID-19 in Manaus, Brazil, shows that a largely unmitigated epidemic can infect a high fraction of the population and cause high mortality.

Brazil has experienced one of the world’s most rapidly growing COVID-19 epidemics, with the Amazon being the worst-hit region (*1*). Manaus is the largest metropolis in the Amazon, with a population of more than 2 million and a population density of 158 inhabitants/km^2^. The first severe acute respiratory syndrome coronavirus 2 (SARS-CoV-2) case in Manaus was confirmed on 13 March 2020 (*2*) and was followed by an explosive epidemic, peaking in early May with 4.5-fold excess mortality (*3*). This was followed by a sustained drop in new cases despite relaxation of nonpharmaceutical interventions (NPIs). The prevalence of antibodies to SARS-CoV-2 is an estimate of the attack rate in Manaus and provides a data-based estimate of the extent of COVID-19 spread in the absence of effective mitigation.

Given a basic reproduction number (*R*_0_) of 2.5 to 3.0 for Amazonas state (*4*), the expected attack rate during an unmitigated epidemic in a homogeneously mixed population is 89 to 94% (*5*). When the percentage of infected people exceeds the herd immunity threshold of 60 to 67%, or 100 × [1 – (1/*R*_0_)], each infection generates fewer than one secondary case (case reproduction number *R*_t_ < 1) and incidence declines. We sought to measure the SARS-CoV-2 attack rate in Manaus and to explore whether the epidemic was contained (*R*_t_ < 1) because infection reached the herd immunity threshold, or because of other factors such as behavioral changes and NPIs. We compared data from Manaus with findings from São Paulo, where the first Brazilian COVID-19 cases were detected (*2*, *6*) and both the rise and fall in mortality were slower and more protracted.

We used a chemiluminescent microparticle immunoassay (CMIA; AdviseDx, Abbott) that detects immunoglobulin G (IgG) antibodies to the SARS-CoV-2 nucleocapsid (N) protein. To infer the attack rate from antibody test positivity, we need to account for the sensitivity and specificity of the test (*7*). The specificity of the CMIA is high (>99.0%) (*8*–*10*), but previous high (>90.0%) sensitivity estimates (*8*, *10*) may not apply to blood donor screening (*11*, *12*) for two reasons. First, most SARS-CoV-2 infections in blood donors are asymptomatic, and weaker antibody responses in asymptomatic disease (*13*) may lead to a lower initial seroconversion rate (i.e., more “serosilent” infections). Second, as a result of antibody waning, sensitivity falls over time (*14*), such that test positivity increasingly underestimates the true attack rate.

**Fig. 1:**
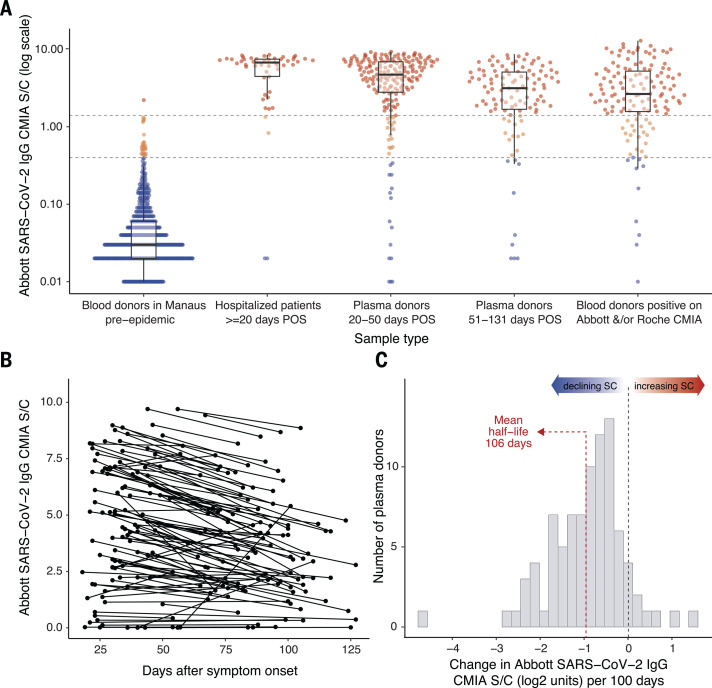
Abbott SARS-CoV-2 N IgG chemiluminescence assay performance and antibody dynamics in different clinical samples. (**A**) Signal-to-cutoff (S/C) values using the Abbott chemiluminescence assay (CMIA) in the following clinical samples (from left to right): 821 routine blood donation samples from Manaus in February 2020, >1 month before the first notified case in the city; 49 samples collected at 20 to 33 days after symptom onset from SARS-CoV-2 PCR-positive patients in São Paulo requiring hospital care; 193 patients in São Paulo with PCR-confirmed symptomatic COVID-19 not requiring hospital care, with plasma donation samples taken in the early convalescent period; 107 samples from the same nonhospitalized plasma donor cohort from the late convalescent period; 133 samples that tested positive on either the Abbott CMIA or the Roche Elecsys assay out of 1000 routine blood donations collected in July 2020 and tested in parallel from the Fundação Pró-Sangue blood center (São Paulo). Upper dashed line denotes the manufacturer’s threshold for positive result of 1.4 S/C; lower dashed line denotes an alternative threshold of 0.4 S/C. In the box plots of Abbott IgG CMIA S/C, the central line is the median; upper and lower hinges are the 25th and 75th centiles, respectively; whiskers show the range, extending to a maximum of 1.5 times the interquartile range from the hinge. (**B**) S/C values of the Abbott CMIA for 104 convalescent plasma donors who were sampled at two different times. (**C**) Histogram of the slopes among 88 individuals shown in (B) who tested positive (>1.4 S/C) at the first time point. POS, post–onset of symptoms.

We used a variety of clinical samples at different time points to gain insight into the dynamics of the anti-N IgG detected by the Abbott CMIA (Fig. 1). In samples from hospitalized COVID-19 patients collected at 20 to 33 days after symptom onset, reflecting high disease severity and optimal timing of blood collection, sensitivity was 91.8% [95% confidence interval (CI), 80.8% to 96.8%], which suggests that ~8% of severe convalescent cases do not develop detectable antibodies. Among a cohort of symptomatic cases with mild disease also tested in the early convalescent period, sensitivity fell to 84.5% (95% CI, 78.7% to 88.9%), indicating that initial seroconversion is lower in milder cases. In samples drawn later (50 to 131 days) from the same mild disease cohort, sensitivity was lower still (80.4%; 95% CI, 71.8% to 86.8%), reflecting antibody waning. Indeed, in a subset of 104 patients with two consecutive blood draws, the signal-to-cutoff (S/C) declined over the period observed (Fig. 1B) and among 88 individuals with a positive reading at the first time point, the mean rate of decay was –0.9 log_2_ S/C units every 100 days (95% CI, –1.1 to –0.75), equating to a half-life of 106 days (95% CI, 89 to 132 days) (Fig. 1C).

Finally, we tested 1000 blood donations given in São Paulo in July 2020 in parallel, using a second high-specificity [>99.0% (*15*)] immunoassay less prone to antibody waning (*14*) (Roche Elecsys). Of these, 103 samples were positive using the Abbott CMIA and an additional 30 were positive using the Roche assay. Assuming that all 133 samples were true positives, the sensitivity of the Abbott N IgG assay was 77.4% (95% CI, 69.6% to 83.7%) on asymptomatic blood donor samples. Samples in July were donated 4 months into the ongoing epidemic in São Paulo; accordingly, the false negatives using the Abbott assay include cases that did not initially seroconvert, as well as past infections that had subsequently seroreverted.

Because specificity was high, with only one false positive result in 821 pre-epidemic donations from Manaus (Fig. 1A), we also attempted to improve assay performance by reducing the threshold for a positive result from 1.4 S/C (as per the manufacturer) to 0.4 S/C. This resulted in 27 false positives and a specificity of 96.7% but substantially improved sensitivity at this threshold (Fig. 1A and table S1).

To estimate the proportion of the population with IgG antibodies to SARS-CoV-2, we used a convenience sample of routine blood donations made at the Fundação Pró-Sangue blood bank in São Paulo and the Fundação Hospitalar de Hematologia e Hemoterapia do Amazonas (HEMOAM) in Manaus. The monthly sample size and sampling dates, spanning February to October, are shown in table S2.

**Fig. 2:**
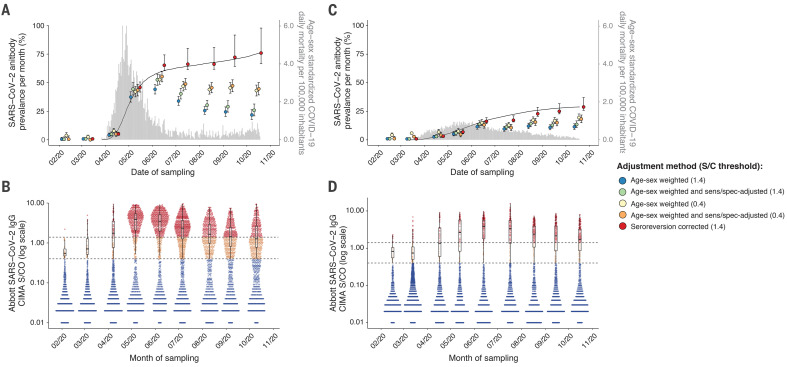
Monthly antibody prevalence and signal-to-cutoff (S/C) reading in Manaus and São Paulo. (**A** and **C**) SARS-CoV-2 antibody prevalence estimates in Manaus (A) and São Paulo (C) with a range of corrections, from left to right: reweighting positive tests, at positivity threshold of 1.4 S/C, to the age and sex distribution of each city; further correcting for sensitivity and specificity at this assay threshold; reweighting positive tests for age and sex at a reduced threshold of 0.4 S/C; correcting for sensitivity and specificity at this threshold; and finally correcting for seroreversion. Error bars are 95% confidence intervals. Gray bars are standardized daily mortality using confirmed COVID-19 deaths from the SIVEP-Gripe (Sistema de Informação de Vigilância Epidemiológica da Gripe; https://covid.saude.gov.br/) notification system and standardized by the direct method using the total projected Brazilian population for 2020 as reference. Black lines are rescaled cumulative deaths, such that the maximum is set to the maximum seroprevalence estimate for each city. Mortality data are plotted according to the date of death. (**B** and **D**) Distribution of S/C values over the nine monthly samples are shown for Manaus (B) and São Paulo (D). Each point represents the S/C reading for a single donation sample. Upper dashed line denotes the manufacturer’s threshold (1.4 S/C units); lower dashed line denotes an alternative threshold (0.4 S/C units); black box plots show the median (central lines), interquartile range (hinges), and range extending to 1.5 times the interquartile range from each hinge (whiskers) of S/C values above 0.4 (i.e., excluding very low and likely true-negative values).

The prevalence of SARS-CoV-2 antibodies in February and March was low (<1%) in both São Paulo and Manaus. This is consistent with the timing of the first confirmed cases that were diagnosed on 13 March in Manaus and on 25 February in São Paulo (*2*). In Manaus, after adjustment for the sensitivity and specificity of the test (but not antibody waning) and reweighting for age and sex, the prevalence of SARS-CoV-2 IgG antibodies was 4.8% (95% CI, 3.3% to 6.8%) in April and 44.5% (95% CI, 39.2% to 50.0%) in May, reaching a peak of 52.5% (47.6% to 57.5%) in June (Fig. 2 and table S2). The increasing seroprevalence closely followed the curve of cumulative deaths. In São Paulo, the prevalence of SARS-CoV-2 IgG in blood donors also increased steadily, reaching 13.6% (95% CI, 12.0% to 8.1%) in June.

Between June and October, the effect of seroreversion became apparent in both cities. In Manaus, after the peak antibody prevalence in June, the proportion of blood donors who tested positive fell steadily to 25.8% in October. Excluding extreme negative samples (<0.4 S/C), the median assay signal fell steadily from May: 3.9 (May), 3.5 (June), 2.3 (July), 1.7 (August), 1.4 (September), and 1.3 (October) (Fig. 2B). Similarly, in São Paulo, antibody prevalence remained stable between June and October while the number of daily COVID-19 deaths also remained relatively stable, reflecting a balance between antibody waning from infections earlier in the outbreak and seroconversions following recent infections (Fig. 2C).

In Manaus, the effect of antibody waning on apparent prevalence was partially ameliorated by reducing the threshold for a positive result from 1.4 S/C to 0.4 S/C and correcting for the resulting increased false positive rate. However, the results in São Paulo were largely unchanged by this correction (Fig. 2 and table S2).

We further corrected for seroreversion with a model-based approach (see supplementary materials). Briefly, we assumed that the probability of an individual seroreverting exactly *m* months after recovery decays exponentially with *m*. We estimated the decay rate and the proportion of patients who seroreverted using the seroprevalence data from Manaus to find the decay rate that minimized the number of new cases in July and August while avoiding decreases in prevalence—that is, assuming there were few cases in Manaus in July and August and that changes in seroprevalence were due mainly to waning antibodies. The results of these corrections are shown in Fig. 2 and table S2. After adjusting for seroreversion, we find that cumulative incidence in Manaus may have reached as high as 66.2% (95% CI, 61.5% to 80.1%) in July and 76.0% (95% CI, 66.6% to 97.9%) in October. The reliability of this estimate depends on the validity of the exponential decay assumption, and in the absence of an accepted approach to account for seroreversion, these results should be interpreted with caution.

To calculate infection fatality ratios (IFRs), we used the prevalence (adjusted for sensitivity and specificity, and reweighted for age and sex) in June, as this followed the epidemic peak in Manaus but preceded appreciable seroreversion. In Manaus, the IFRs were 0.17% and 0.28%, taking into consideration the numbers of polymerase chain reaction (PCR)–confirmed COVID-19 deaths and probable COVID-19 deaths based on syndromic identification, respectively. In São Paulo, the global IFRs were 0.46% and 0.72%, respectively. The difference may be explained by an older population structure in São Paulo (fig. S1A). Supporting this inference, the age-specific IFRs were similar in the two cities, and were similar to estimates based on data from China (*16*) (fig. S1B) and a recent systematic review (*17*). We also obtained similar age-specific IFRs using the seroreversion-corrected prevalence estimates from October (fig. S1).

Blood donors may not be representative of the wider population. In both cities, the eligible age range for blood donation in Brazil (16 to 69 years) and the sex distribution of donors are different from those of the underlying population (fig. S2). Reweighting our estimates for age and sex (Fig. 2 and table S2) resulted in a slight reduction in prevalence, particularly in Manaus, where men were overrepresented among donors and also had a higher seroprevalence (fig. S3). Self-reported ethnicity in donors was similar to that of the census populations (fig. S2). The median income in blood donors’ census tracts of residence was marginally higher than a population-weighted average for both cities (fig. S4). Regarding the spatial distribution of donors, there was a similar antibody prevalence across different regions sampled in both cities (fig. S5), and we achieved good geographic coverage in both cities (see supplementary materials and fig. S5).

Because potential donors are deferred if they have a positive SARS-CoV-2 PCR test or clinical diagnosis of COVID-19, increasing access to testing might have reduced the pool of eligible donors through time. However, only 2.7% of residents in Manaus and 8.5% in São Paulo reported having a PCR test performed by September (fig. S6). As such, changing access to testing is unlikely to have been important. Considering these factors together, we suggest that our results can be cautiously extrapolated to the population aged 16 to 69 years in Manaus and São Paulo. Within this group, studies of blood donors may underestimate the true exposure to SARS-CoV-2 because donors may have higher socioeconomic profiles and greater health awareness and engagement, and because symptomatic donors are deferred. However, it is likely that seroprevalence in children and older adults is lower.

Our results show that between 44% and 66% of the population of Manaus was infected with SARS-CoV-2 by July, following the epidemic peak there. The lower estimate does not account for false negative cases or antibody waning; the upper estimate accounts for both. *R*_t_ fell to <1 (fig. S7) in late April when cumulative infections were between 5% and 46% of the population. NPIs (table S3) were implemented in mid- to late March when physical distancing also increased (fig. S8). It is likely that these factors worked in tandem with growing population immunity to contain the epidemic. Transmission has since continued in Manaus, albeit to a lesser extent than in April and May (Fig. 2 and fig. S7). From the second week of August there has been a small increase in the number of cases (*18*), which, at the time of writing, has begun to decline. Consequently, the attack rate rose to 76% in October. This remains lower than predicted in a homogeneously mixed population with no mitigation strategies (~90%). Homogeneous mixing is unlikely to be a valid assumption (*19*), and behavioral change and NPIs may explain why the estimated final epidemic size has not yet reached 89 to 94%, as expected for *R*_0_ values between 2.5 and 3.0 (*4*).

By 1 October, Manaus recorded 2642 [1193/million inhabitants (mil)] COVID-19 confirmed deaths and 3789 (1710/mil) severe acute respiratory syndrome deaths; São Paulo recorded 12,988 (1070/mil) and 20,063 (1652/mil), respectively. The cumulative mortality proportions were similar in both cities and high relative to other locations such as the United Kingdom (620/mil), France (490/mil), or the United States (625/mil) as of 1 October (*20*). The different attack rates in Manaus and São Paulo (76% versus 29% of people infected), despite similar overall mortality rates, are due to the higher IFR in São Paulo. The age-standardized mortality ratio was 2.0 comparing observed deaths in Manaus to those expected from projecting the age-specific mortality in São Paulo onto the age structure of Manaus. The *R*_0_ was similar in the two cities (fig. S7), but cases and deaths increased and then decreased more slowly in São Paulo than in Manaus where both the rise and fall were more abrupt (fig. S7). The lower attack rate in São Paulo is partly explained by the larger population size (2.2 million versus 12.2 million inhabitants). As population size increases, the time to reach a given attack rate also increases (*21*).

The attack rate in Manaus is higher than estimates based on seroprevalence studies conducted in Europe and North America (*8*, *22*, *23*) and on recent results from Kenyan blood donors (*24*). A similarly high seroprevalence (~50%) was observed in slums in Mumbai, India (*25*). In Brazil, one population-based serosurvey in São Paulo (*26*) found a prevalence similar to that in our study (26.2% versus 28.8% in blood donors, in October). In Manaus, a lower seroprevalence (14%, in June) was found in a random household sample of 250 people (*1*). But this study was not powered at the city level and used the lower-sensitivity Wondfo (*27*) rapid test. As such, the results are not directly comparable.

Future investigations should be conducted to determine what accounted for such extensive transmission of SARS-CoV-2 in Manaus. Possible explanations include socioeconomic conditions, household crowding (*28*), limited access to clean water, and reliance on boat travel (*1*) in which overcrowding results in accelerated contagion, similar to that seen on cruise ships (*29*). The young mobile population with potentially low preexisting immunity to SARS-CoV-2 (*30*), as well as the early circulation of multiple virus lineages introduced from multiple locations, may have contributed to the large scale of the outbreak.

Our data show that >70% of the population had been infected in Manaus about 7 months after the virus first arrived in the city. This is above the theoretical herd immunity threshold. However, prior infection may not confer long-lasting immunity (*30*, *31*). Indeed, we observed rapid antibody waning in Manaus, consistent with other reports that have shown signal waning on the Abbott IgG assay (*14*, *32*). However, other commercial assays, with different designs or targeting different antigens, have more stable signal (*14*), and there is evidence for a robust neutralizing antibody response several months out from infection (*33*). Rare reports of reinfection have been confirmed (*34*), but the frequency of its occurrence remains an open question (*35*). Manaus represents a “sentinel” population, giving us a data-based indication of what may happen if SARS-CoV-2 is allowed to spread largely unmitigated. Further seroepidemiological, molecular, and genomic surveillance studies in the region are required urgently to determine the longevity of population immunity, the correlation with the observed antibody waning, and the diversity of circulating lineages. Monitoring of new cases and the ratio of local versus imported cases will also be vital to understand the extent to which population immunity might prevent future transmission, and the potential need for booster vaccinations to bolster protective immunity.

## Supplementary Materials

science.sciencemag.org/content/371/6526/288/suppl/DC1 Materials and Methods Figs. S1 to S9 Tables S1 to S3 References (*37*–*41*) MDAR Reproducibility Checklist

## Appendix

View/request a protocol for this paper from *Bio-protocol*.
